# Trace quantification of GL-V9 and its glucuronide metabolites (5-O-glucuronide GL-V9) in Beagle dog plasma by UPLC–MS/MS and its application to a pharmacokinetic study

**DOI:** 10.1371/journal.pone.0286467

**Published:** 2023-06-07

**Authors:** Xuefeng Zhang, Guanlan Liu, Zechun Sang, Qinglong Guo, Yuxin Zhou

**Affiliations:** 1 State Key Laboratory of Natural Medicines, Jiangsu Key Laboratory of Carcinogenesis and Intervention, Department of Physiology, School of Basic Medicine and Clinical Pharmacy, China Pharmaceutical University, Nanjing, People’s Republic of China; 2 TriApex Laboratories Co., Ltd, Nanjing, People’s Republic of China; Universita degli studi della Campania, ITALY

## Abstract

GL-V9, a new synthetic flavonoid derived from wogonin, has shown beneficial biological functions. In this study, accurate and sensitive UPLC–MS/MS methods were developed and validated for the quantification of GL-V9 and its glucuronide metabolite (5-O-glucuronide GL-V9) in Beagle dog plasma. The chromatographic separation was performed on a C_8_ column (ACE Excel 5 C_8_ 50×3.0 mm) using 0.1% formic acid and acetonitrile were used as mobile phase. Mass detection was performed on a triple quadrupole tandem mass spectrometer equipped with an electrospray ionization (ESI) interface operating in positive ion mode. Quantitative analysis was performed in multiple reaction monitoring (MRM) mode with the transitions of *m/z* 410.2→126.1 for GL-V9, *m/z* 586.3→410.0 for 5-O-glucuronide GL-V9 and *m/z* 180.0→110.3 for phenacetin (internal standard), respectively. The calibration curves for GL-V9 and 5-O-glucuronide GL-V9 showed excellent linearity over the concentration range of 0.5–500 ng/mL with correlation coefficient greater than 0.99. The intra- and inter-day accuracies were within 99.86% to 109.20% for GL-V9 and 92.55% to 106.20% for 5-O-glucuronide GL-V9, respectively. The mean recovery was 88.64% ± 2.70% for GL-V9, and 92.31% ± 6.28% for 5-O-glucuronide GL-V9, respectively. The validated method was successfully applied to the pharmacokinetic study in Beagle dogs after oral and intravenous administration. The oral bioavailability of GL-V9 was approximately 2.47%~4.35% in Beagle dogs and reached steady state on the fifth day after repeated dosing.

## Introduction

Cancer, the abnormal growth of cells, is an enigmatic and frightening disease or group of diseases that places an enormous burden on society worldwide. Cancer occurs with the involvement of a variety of factors, genes, and pathways and shows multiple stages [1, 2]. In recent years, advances such as exploring molecular signaling pathways and modulating the cellular microenvironment have improved treatments of various cancer. Much effort is still needed to be done to discover and develop innovative drugs involved in various anticancer mechanisms.

**Fig 1:**
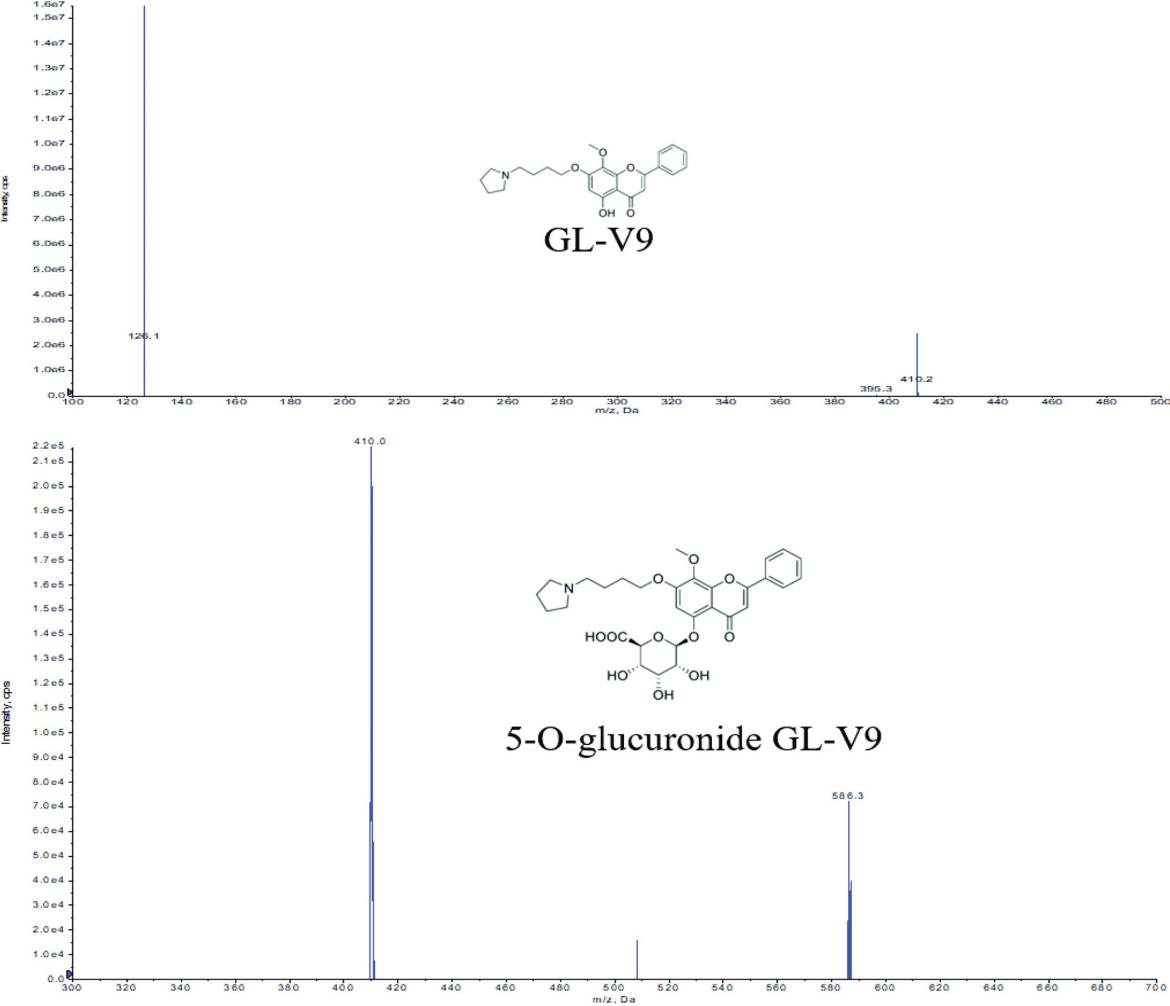
Mass spectrum of analyte (Q1 & Q3 mass spectrum of GL-V9 and 5-O-glucuronide GL-V9).

5-hydroxy-8-methoxy-7-(4-(pyrrolidin-1-yl) butoxy)-4 Hchromen-4-one, (GL-V9, Fig 1), is composed of a typical chemical structure of flavonoids, with two benzene rings (A and B) connected by an oxygen containing heterocyclic ring (C). Numerous preclinical studies have provided sufficient evidence to suggest that flavonoids may be important adjuvants in cancer therapy [3]. In addition, the previous study reported that GL-V9 played several important roles in anti-cancer effect. Research had shown that GL-V9 could affect PI3K/Ak and MMP-2/9 axis to suppress CRC cell migration and invasion [4], disrupt mitochondrial binding of HKII and induce apoptosis and reduced glycosylation in breast cancer cells [5], eliminate drug-induced senescent MEFs (Mouse embryonic fibroblasts) and senescent breast cancer cells [6], upregulate expression of Trx-1 through activation of the AMPK/FOXO3a pathway and ameliorate the effect of DSS-induced colitis on oxidative stress [7], induces p53 associated senescence and catastrophic mitosis in malignant T cells at sublethal doses [8] and inhibits the expression and nuclear translocation of Trx-1, followed by inhibition of the DNA binding activity of HIF-1α, by suppressing the Trx-1/Ref-1 axis [9], i.e. GL-V9 exhibited extensive anti-tumour mechanisms, including anti-tumor immunity, redox metabolism, cell proliferation, autophagy, apoptosis, cell cycle, and so on. On the other hand, for flavonoids, due to the presence of extensive hydroxy groups, phase II metabolism cannot be neglected [10]. The literature had shown that GL-V9 showed extensive metabolism and the 5-O-glucuronide GL-V9 is the only glucuronide metabolite and the dominant product of GL-V9 phase II metabolism *in vivo and in vitro* in the previous study [11, 12]. Wogonin and its glucuronidation metabolite (wogonoside) have been shown to have immense therapeutic potential against cancer by regulating various cell signaling pathways [13]. It could speculated that GL-V9, a prominent derivative of wogonin, and its glucuronide metabolite (5-O-glucuronide GL-V9) may possess similar anticancer activities. The basic pharmacokinetic characteristics of GL-V9 in rats were investigated after oral and pulmonary administration. Double peaks were observed due to the presence of enterohepatic after oral administration and the bioavailability was about 8.54% in rat [11]. These proven pharmacodynamic and pharmacokinetic properties of GL-V9 are just the tip of the iceberg, and much more work further needs to be done on GL-V9. Meanwhile, we should explore the characteristics of the its glucuronide metabolite (5-O-glucuronide GL-V9). Therefore, a comprehensive understanding on the pharmacokinetic of GL-V9 and its glucuronide metabolite after administration for their best anticancer efficacy is an emergency. In this study, we illustrated the characteristics of GL-V9 and its glucuronide metabolite (5-O-glucuronide GL-V9) after administration of single dose and repeated doses of GL-V9 in Beagle dogs. However, to the best of our knowledge, there is no report on the pharmacokinetics of GL-V9 and its glucuronide metabolite (5-O-glucuronide GL-V9) using sensitive and reproducible analytical methods in Beagle dog plasma. There are few references reporting the detection of GL-V9 in rat plasma, without mentioning 5-O-glucuronide GL-V9. Due to its high sensitivity and selectivity, UPLC–MS/MS has been widely used for quantitative analysis in biological samples. Therefore, we attempted to develop UPLC–MS/MS methods for the detection GL-V9 and its glucuronide metabolite (5-O-glucuronide GL-V9) and fully validated the developed methods.

In this study, the sensitive and reliable UPLC–MS/MS methods were established. In addition, the successfully validated bioanalytical methods were applied for the first time to the post-ingestion pharmacokinetics of GL-V9 in Beagle dogs.

## 2. Materials and methods

### 2.1. Chemicals and reagents

GL-V9 (MW 409.48, purity 98.48%, Fig 1) and 5-O-glucuronide GL-V9 acetate (MW 645.65, NMR purity 88.00%, Fig 1) were synthesized by China Pharmaceutical University [14]. Phenacetin (internal standard, purity 98.77%) was purchased from Dr. Ehrenstorfer GmbH Company (Augsburg, Germany). Methanol and acetonitrile (HPLC grade) were purchased from Fisher Scientific Company. Formic acid (LC-MS grade) was purchased from MACKLIN Company and DMSO (HPLC grade) was purchased from SIGMA Company. The ultrapure water used for UPLC–MS/MS was prepared from an Elix-Milli-Q water purification system (Millipore, Bedford, USA). All other chemicals were of analytical grade.

### 2.2. Instrument and analytical conditions

The LC system was Shimadzu EXion LC-AD ^TM^ system equipped with a cooling auto-sampler and column oven. Chromatographic separation was carried out on an ACE Excel 5 C_8_ column (50×3.0 mm) kept at a constant temperature of 40°C. The mobile phase was composed of 0.1% formic acid (A) and acetonitrile (B). The flow rate was set at 0.5 mL/min. The optimized gradient elution programs were as follows: 43% B at 0–1.3 min, 43–85% B at 1.3–1.5 min, 85% B at 1.5–2 min and 43% B at 2–3 min for GL-V9; 30% B at 0–0.2 min, 30–60% B at 0.2–0.7 min, 60% B at 0.7–1.6 min, 60–30% B at 1.6–1.7 min and 30% B at 1.7–2.5 min for 5-O-glucuronide GL-V9. The auto-sampler temperature was set at 4°C.

Mass detection was obtained on an AB SCIEX triple-quadrupole mass spectrometer (TRIPLE QUAD 5500, SCIEX) equipped with a turbo spray ionization operating in positive ionization mode. The detection of the ions was carried out in MRM mode with the transitions of *m/z* 410.2→126.1 for GL-V9, *m/z* 586.3→410.0 for 5-O-glucuronide GL-V9) and *m/z* 180.0→110.3 for IS, respectively. The interface heater was on and nitrogen was used in all cases. The ion source parameters were optimized as following: ion spray voltage was all 5000 V; collision energy were 28 V for GL-V9, 19 V for 5-O-glucuronide GL-V9 and 40 V for IS, respectively. Data acquisition were performed using Analyst software (version 1.7.0 package).

### 2.3. Preparation of working solution, standard and quality control samples

The stock solutions of GL-V9, 5-O-glucuronide GL-V9 and IS all at 1000 ngg/mL were individually prepared by dissolving the reference standards into DMSO. Final concentrations of IS (Phenacetin) working solution by dissolving stock solution in acetonitrile were 20 ng/ml and 100 ng/ml, respectively.

The working solutions of GL-V9 and 5-O-glucuronide GL-V9 were prepared by serially diluting the stock solutions using 50% acetonitrile. Calibration standards for GL-V9 and 5-O-glucuronide GL-V9 were freshly prepared by spiking 10 μL working solution into 90 μL of blank Beagle dog plasma. And the volume can be adjusted proportionally to keep the final concentration consistent. Terminal concentrations of GL-V9 and 5-O-glucuronide GL-V9 in Beagle dog plasma samples were both 0.5,1,2,10,50,150, 300 and 500 ng/mL.

Lower limit of quantitation (LLOQ) and quality control (QC) samples were also prepared in the same manner at the concentration of 0.5, 1.5, 20, 200 and 400 ng/ml for both. All the solutions were stored in the freezer at -20°C and brought to room temperature before use.

### 2.4. Sample pre-treatment

A plasma protein precipitation method was applied to extract GL-V9 or 5-O-glucuronide GL-V9 from Beagle dog plasma.

For the extraction of GL-V9, an aliquot of 20 μL of plasma sample was drawn into the EP tubes and then 400 μL of acetonitrile (containing 100 ng/mL IS) were added to each tube. The mixture was vortexed for 3 min at 600 g and then centrifuged for 5 min at 13400 g (at 4°C). Afterwards, an aliquot of 20 μL of the supernatant was mixed with 480 μL of solvent (50% acetonitrile and 50% water). An aliquot of 5 μL was submitted for analysis.

For the extraction of 5-O-glucuronide GL-V9, an aliquot of 20 μL of plasma sample was drawn into the tube, and then 100 μL of acetonitrile (containing 20 ng/mL IS) was added to each tube. The mixture was vortexed for 3 min at 600 g and centrifuged for 5 min at 13400 g (at 4°C). Afterwards, an aliquot of 80 μL of the supernatant was mixed evenly with 90 μL of ultrapure water. An aliquot of 2 μL was submitted for analysis.

### 2.5. Method development and validation

Bioanalytical methods were well characterized and validated to ensure reliable data. Specificity, matrix effects, calibration curve, LLOQ, precision and accuracy, recovery and stability and reinjection reproducibility were validated as described below according to the “Bioanalytical Method Validation Guidance for Industry” (FDA, 2018) and “Bioanalytical Method Validation M10 (Draft)” (ICH, 2019).

#### 2.5.1. Selectivity

Blank plasma samples obtained from six individuals should be evaluated to investigate any potential interference of analyte and internal standard. Besides, the interfere between of the internal standard and analytes was investigated. The selectivity sample (I-BK) obtained from mixed blank plasma will be processed in accordance with the pretreatment methods of BK samples that were spiked with no analyte but internal standard to evaluate the interference of the internal standard to the analyte. The selectivity sample (I-ULOQ) at the concentration of ULOQ obtained from the mixed blank plasma that spiked with no internal standard but analytes will be processed to evaluate the interference of analyte to the internal standard. Three replicates were processed and analyzed in parallel. The selectivity was measured by comparing the MRM chromatograms of the blank plasma with those of the blank plasma sample spiked with analytes.

#### 2.5.2. Calibration curve and LLOQ

The calibration curves were generated by plotting the peak area ratio of the analytes to that of IS against the nominal concentration of the calibration standards (0.5,1,2,10,50,150, 300 and 500 ng/mL). Linearity was evaluated through Watson LIMS 7.4 with the weighted (W = 1/x^2^) least squares regression method. The calibration curves had to have a correlation coefficient (*r*) of 0.99 or better. The LLOQ was defined as the lowest of the concentration of the calibration curve at which the signal-to-noise ratio should be at least 10 and the precision should not exceed 20% and the accuracy was within ± 20%.

#### 2.5.3. Precision and accuracy

The precision and accuracy were assessed by spiking blank Beagle dog plasma with known concentrations (LLOQ: 0.5 ng/mL, QC 1:1.5 ng/mL, QC 2:20 ng/mL, QC 3: 200 ng/mL, QC 4: 400 ng/mL) of GL-V9 or 5-O-glucuronide GL-V9 on three continuous days. At each concentration level, six replicate samples were prepared.

Coefficient of variation (CV%) was used to evaluate the intra- and inter-day precision, which should be below 15%, except at LLOQ where the RSD should not exceed 20%. The accuracy was calculated using the following equation: Accuracy (%) = Measured concentration/Nominal concentration*100. The value of accuracy (%) should be in the range of 85–115%, except at LLOQ where the value should be within 80–120%.

#### 2.5.4. Recovery and matrix effect

The recovery of GL-V9 and 5-O-glucuronide GL-V9 was determined by comparing the peak areas of the regularly prepared QC samples with those of the post-extracted blank samples spiked at the identical concentration (six replicates at each concentration level).

Preparation of matrix containing samples: Blank plasma from six individuals were processed in accordance with the processing method of DBK samples with six replicates of each individual, of which three samples were added with the QC1-Neat solution and three samples were added with the QC4-Neat solution. Preparation of matrix free samples: Ultrapure water were used instead of blank plasma followed by the same treatment steps as Matrix Containing samples above with six replicates. For each individual sample, the ratio of the analyte mean peak area in matrix containing samples to the analyte mean peak area in matrix free samples is served as the matrix factor (MF) of analyte, and the ratio of the internal standard mean peak area in matrix containing samples to the internal standard mean peak area in Matrix Free samples is served as the MF of internal standard. The ratio of the analyte MF to the internal standard MF is served as the internal standard normalized MF.The precision (%CV) of internal standard normalized MF from six individuals should not exceed 15% at two concentration levels (QC1 and QC4), respectively.

#### 2.5.5. Stability

The stability was investigated by analyzing three replicates of the plasma samples at different concentration levels under various conditions including sample preparation stability (at room temperature for at least 8h), processed sample stability (at 4°C for at least 24 h), freeze-thaw circles (freeze at -65~-90°C and thaw at room temperature) and long term (stored at -65~-90°C for at least 30 h).

The accuracy (Bias%) of the mean value at each concentration level should be within ±15% and the precision (CV%) at each concentration level should be no more than 15%.

Besides sample stability, the reinjection reproducibility, whole blood stability and working and stock solution stability were also investigated.

### 2.6. Pharmacokinetic study

Animal experiment was in accordance with animal welfare policies and guidelines, which was approved by IACUC (The Institutional Animal Care and Use Committee) prior to the use. SPF Beagle dogs, weighted 6~10 kg, were purchased from Yadong Experimental Animal Research Institute Co., Ltd. (Nanjing, China). The dogs were kept in an environmentally controlled breeding room (temperature 19°C ~26°C relative humidity 40–70%, and 12 h on/12 h off light cycle) until experiments started. Animals in this study were monitored weekly for weight changes, signs of discomfort, or poor health; where humanely indicated by such endpoints (e.g., signs of pain, inability to reach food, or ≥ 20% weight loss), and were anesthetized by inhalation of isoflurane and sacrificed by bleeding from the abdominal aorta. All experimental procedures and protocols were approved by the Institutional Animal Care and Use Committee of TriApex Laboratories Co., Ltd. [No. SYXK-(Su)-2020-0007].

A total of twenty-four dogs (each group: six animals, half male and half female) were randomly divided into four groups. The dogs were fasted for at least 12 h before dosing, free access to water.

For oral administration, dogs received a single dose of 30, 90 or 150 mg/kg of GL-V9 formulated in 0.5% CMC-Na solution. The blood samples were collected into tubes containing EDTA-K_2_ at 0, 0.17, 0.5, 1, 2, 4, 8, 12, and 24 h post-dose. For repeated oral administration, GL-V9, formulated in a 0.5% CMC Na solution, was administered to dogs at a single dose of 90 mg/kg every 24 h for a continuous period of 7 days., the samples were collected at 0 hand 4 h pose-dose after the third/forth/fifth/sixth dosing and 0, 0.17, 0.5, 1, 2, 4, 8, 12, and 24 h post-dose on the first and seventh dosing. For intravenous administration, GL-V9 was prepared in a solution containing 50000 ng/mL glucose and 1660 ng/mL lactic acid and administered at a single dose of 3 mg/kg. The blood samples were collected at 0, 0.083, 0.25, 1, 2, 4, 8, 12 and 24 h post-dose. All the blood samples were then centrifuged at 1500 g for 10 min and the plasma fractions were withdrawn and then frozen at -65 ~-90°C until analysis.

### 2.7. Data analysis

Data acquired by the Analyst software were imported into Watson LIMS (v7.4, Thermo Fisher Scientific™) for final regression and pharmacokinetic parameters were estimated by Phoenix WinNonlin 8.1 software (Pharsight®, a Certara™ company, USA) with a non-compartmental model. Data were presented as means ± SD and analyzed by Excel (Microsoft). The values were indicated to be extremely significant different when p <0.01 by applying Student’s t-test.

Oral bioavailability (F) was calculated according to the following equation:

$$F(\%)=(AUC_{ig}/AUC_{iv})\times (Dose_{iv}/Dose_{ig})\times 100\%$$

## 3. Results and discussion

### 3.1. Method development

Glucuronic acid conjugated metabolites could be converted back to the parent drug due to in-source collision-induced dissociation (CID) using LC-MS/MS for detecting [15], therefore, special notice taken to the in-source collision in optimizing the parameters for detecting to minimize the interference of 5-O-glucuronide GL-V9 to GL-V9 in case of in-source CID. Final parameters are shown in the Section 2.2.2. Meanwhile, the responses of GL-V9 and 5-O-glucuronide GL-V9 varied greatly in the UPLC–MS/MS system, we could adjust the adding volume of precipitant and diluent solution to meet the requirement of linearity and instrument response.

At first, we considered simultaneous determination GL-V9 and its glucuronide metabolite (5-O-glucuronide GL-V9) in Beagle dog plasma using one assay method and we actually did it, however, in evaluation of the specify, we found that the interference of 5-O-glucuronide GL-V9 to GL-V9 did not meet the acceptance criteria and when analyzing 5-O-glucuronide GL-V9 alone, we detected GL-V9, linear within a certain of concentration range. GL-V9 and other compounds reacted under the protection of nitrogen to synthetic 5-O-glucuronide GL-V9 [12], that is, the standard of 5-O-glucuronide GL-V9 may mix with GL-V9. And the fact is that the standard of 5-O-glucuronide GL-V9 contains GL-V9 (about 1%, mole fraction), so we had to explore two methods to detect 5-O-glucuronide GL-V9 and GL-V9, respectively. We should choose the same column or mobile phase as far as possible for the convenience of measuring biological samples to avoid the repeated freeze-thaw of the samples or continuously detect samples. Different mobile phases (Phase A and Phase B) or composition of mobile phase were examined and the mobile phase composed of water (containing 0.1% formic acid, A) and acetonitrile (B) obtained efficient chromatography. A C_8_ column (ACE Excel 5 C_8_ 50×3.0 mm) was chosen for its excellent peak symmetry in the similar way. Carryover, changes in measured concentrations due to residual analytes from the previous sample that remain in the analyzer, is a big concern of detecting flavonoid compounds using UPLC–MS/MS. Therefore, residues on the instrument were cleaned by gradient elution, especially for GL-V9.

Overall, using our optimized liquid phase conditions and mass spectrometry conditions, the validated item like precision and accuracy carryover, recovery, matrix effect, etc., all met the analytical requirement.

### 3.2 Method validation

#### 3.2.1 Specificity and selectivity

**Fig 2:**
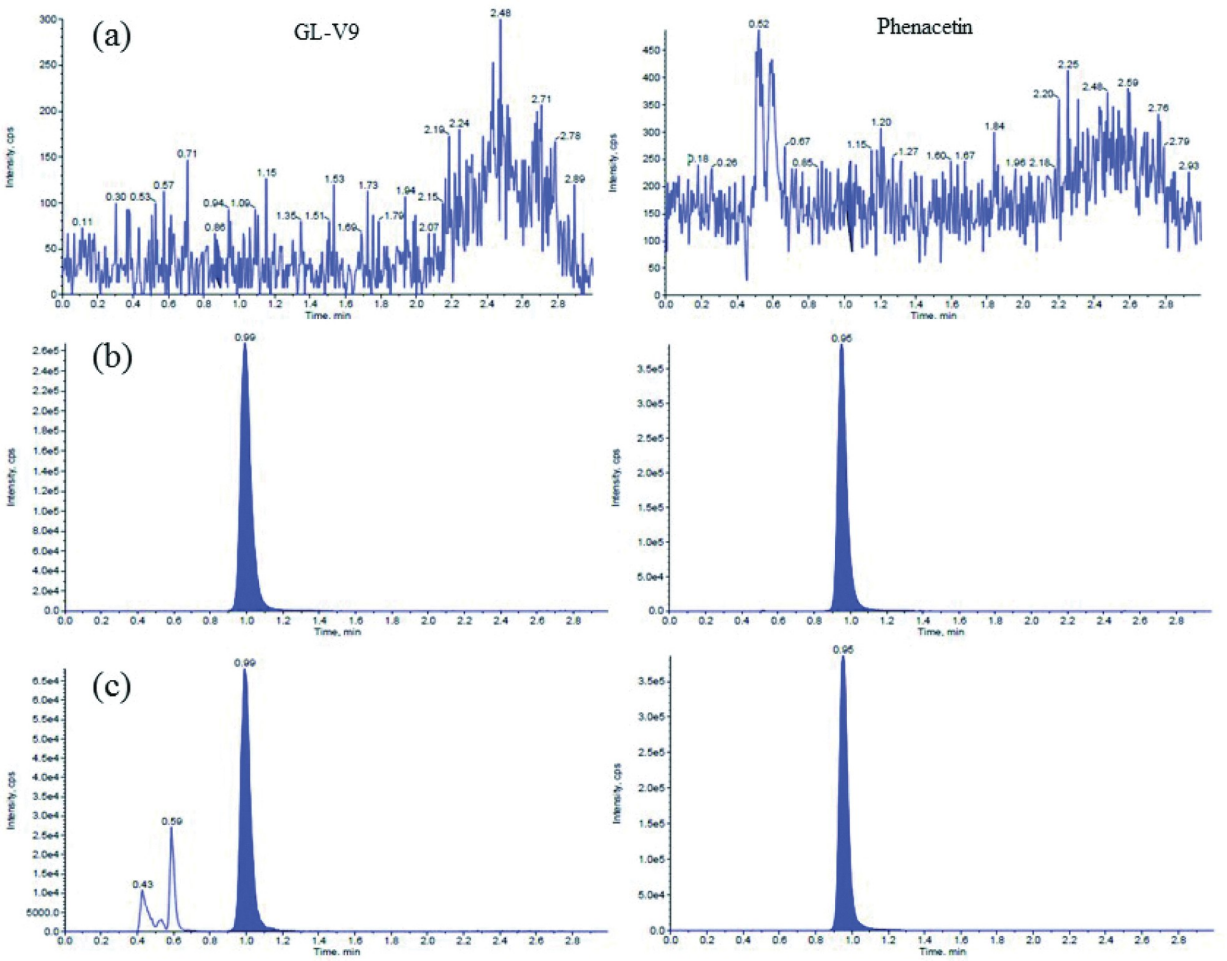
Representative UPLC–MS/MS chromatograms of GL-V9 and IS. (A) blank plasma; (B) calibration standard at 50ng/ml; (c) Beagle plasma sample obtained at 15min after an intravenous administration of GL-V9 at 1.5mg/kg (left standard for GL-V9, right standard for IS).

**Fig 3:**
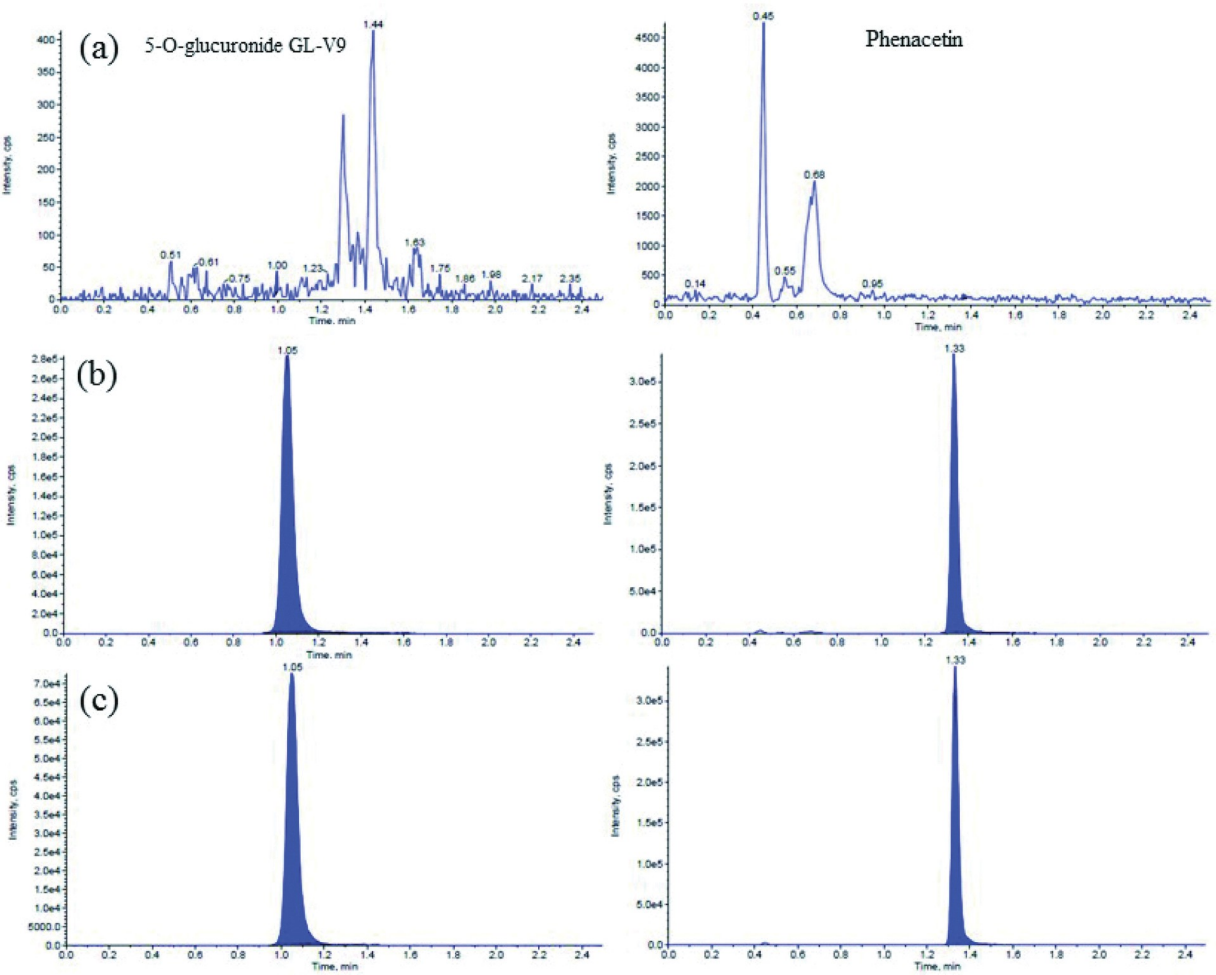
Representative UPLC–MS/MS chromatograms of 5-O-glucuronide-GL-V9 and IS. (A) blank plasma; (B) calibration standard at 50ng/ml; (C) Beagle plasma sample obtained at 15min after an intravenous administration of GL-V9 at 1.5mg/kg (left standard for 5-O-glucuronide-GL-V9, right standard for IS).

Typical MRM chromatograms of blank dog plasma, blank dog plasma spiked with analytes and IS, and plasma sample collected after administration are shown in Figs 2 and 3. As shown in Figs 2 and 3, no endogenous interferences were observed at the retention times of the analytes and IS.

#### 3.2.2 Calibration curve and LLOQ

The calibration curves showed a satisfactory linearity over the corresponding range of 0.5–500 ng/mL for both analytes, with the correlation coefficient more than 0.99 (r > 0.99).

The LLOQ for both analytes was 0.5 ng/mL, at which the signal-to-noise ratio was >10 and the accuracy and precision met the requirements (Table 1).

**Table 1:** Precision and accuracy of GL-V9 and 5-O-glucuronide GL-V9 in Beagle dog plasma.

	Conc. (ng/ml)	GL-V9			5-O-glucuronide GL-V9		
		Mean ± SD (ng/ml)	Accuracy (%)	%CV	Mean ± SD (ng/ml)	Accuracy (%)	%CV
Intra	0.5	0.55 ± 0.04	109.20	7.51	0.53 ± 0.02	105.80	2.84
	1.5	1.53 ± 0.08	101.80	5.24	1.51 ± 0.06	100.53	4.05
	20	20.73 ± 0.60	103.63	2.90	19.38 ± 0.65	96.88	3.34
	200	209.04 ± 3.94	104.52	1.89	191.82 ± 4.72	95.91	2.46
	400	401.63 ± 17.28	100.41	4.30	372.59 ± 15.48	93.15	4.15
Intra	0.5	0.56 ± 0.03	111.40	4.67	0.51 ± 0.02	102.80	4.28
	1.5	1.60 ± 0.08	106.67	5.00	1.56 ± 0.05	104.13	2.88
	20	21.03 ± 1.01	105.14	4.79	19.91 ± 0.45	99.54	2.27
	200	211.21 ± 8.90	105.60	4.21	198.66 ± 4.08	99.33	2.06
	400	405.15 ± 10.03	101.29	2.48	390.33 ± 13.42	97.58	3.44
Intra	0.5	0.53 ± 0.03	105.20	5.70	0.55 ± 0.03	109.80	5.83
	1.5	1.58 ± 0.06	105.00	3.49	1.50 ± 0.09	99.80	6.28
	20	20.36 ± 0.29	101.82	1.40	19.82 ± 0.91	99.10	4.59
	200	204.27 ± 5.77	102.14	2.83	202.11 ± 5.95	101.05	2.94
	400	399.44 ± 5.14	99.86	1.29	370.21 ± 26.71	92.55	7.22
Inter	0.5	0.54 ± 0.03	108.60	6.26	0.53 ± 0.03	106.20	5.08
	1.5	1.57 ± 0.08	104.47	4.79	1.52 ± 0.07	101.47	4.73
	20	20.71 ± 0.71	103.53	3.44	19.70 ± 0.70	98.51	3.53
	200	208.17 ± 6.82	104.09	3.28	197.53 ± 6.42	98.77	3.25
	400	402.07 ± 11.45	100.52	2.85	377.71 ± 20.46	94.43	5.42

#### 3.2.3 Precision and accuracy

The precision and accuracy were assessed at different concentration levels and the results are shown in Table 1. The result suggested that the two methods were accurate and reproducible for the determination of GL-V9 and 5-O-glucuronide GL-V9, respectively, in dog plasma.

#### 3.2.4 Recovery and matrix effect

The precision of internal standard normalization MF (matrix effect factors) at low (QC 1 1.5 ng/mL) and high (QC 4 400 ng/mL) concentration levels were 1.08% and 0.37% for GL-V9, respectively and 4.33% and 4.59% for 5-O-glucuronide GL-V9, respectively. The data suggested there was no significant ion suppression or enhancement for the analytes.

The matrix effect and recovery of GL-V9 and 5-O-glucuronide GL-V9 from dog plasma are presented in Table 2. As shown, the overall precision (CV%) of GL-V9 and IS (Phenacetin, 100 ng/mL) recovery rate was 3.04% and 2.93%, respectively, and the CV% of 5-O-glucuronide GL-V9 and IS (Phenacetin, 20 ng/mL) were 6.81% and 2.91%, respectively. These data suggested that the recovery for GL-V9 and 5-O-glucuronide GL-V9 was constant, demonstrating good accuracy and repeatability of the validated methods.

**Table 2:** Matrix effect and recovery and of GL-V9, 5-O-glucuronide GL-V9 and internal standard (Phenacetin)in Beagle dog plasma (n = 6).

Matrix effect	Conc. (ng/mL)	Individual		GL-V9									5-O-glucuronide GL-V9					
				1#		2#		3#	Accuracy (%)		Precision (%CV)		1#	2#	3#	Accuracy (%)		Precision (%CV)
	1.5	A		1.78		1.69		1.71	14.98		2.56		1.55	1.51	1.57	2.89		1.87
		B		1.73		1.70		1.72	14.47		0.92		1.63	1.70	1.72	11.91		2.83
		C		1.72		1.73		1.70	14.56		0.87		1.62	1.63	1.77	11.64		4.84
		D		1.65		1.68		1.82	14.31		5.19		1.62	1.75	1.73	13.40		4.11
		E		1.73		1.69		1.62	12.22		3.33		1.54	1.64	1.68	8.16		4.33
		F		1.79		1.66		1.72	14.89		3.75		1.69	1.72	1.75	14.56		1.89
	400	A		449.41		449.97		442.23	11.80		0.97		384.11	388.08	386.18	-3.47		0.51
		B		449.97		439.85		449.97	11.65		1.31		421.70	414.02	407.45	3.60		1.72
		C		457.83		445.63		444.58	12.34		1.64		424.25	417.99	424.84	5.59		0.90
		D		442.74		445.63		452.60	11.75		1.13		428.55	427.35	433.57	7.46		0.77
		E		438.40		447.08		447.08	11.05		1.13		417.27	419.14	417.57	4.50		0.24
		F		444.19		451.13		442.74	11.50		1.01		446.23	448.77	436.94	11.00		1.40
Recovery	Conc. (ng/mL)		GL-V9				IS (Phenacetin,100 ng/mL)					5-O-glucuronide GL-V9					IS (Phenacetin, 20 ng/mL)	
			Mean ± SD (%)		%CV		Mean ± SD (%)			%CV		Mean ± SD (%)			%CV	Mean ± SD (%)		%CV
	1.5		88.38 ± 2.05		2.32		93.86 ± 2.72			2.90		89.93 ± 6.00			6.67	100.47 ± 2.19		2.18
	200		90.95 ± 2.29		2.52		94.83 ± 2.07			2.19		94.05 ± 3.54			3.76	103.27 ± 2.43		2.35
	400		86.61 ± 1.95		2.25		93.78 ± 3.65			3.89		92.93 ± 8.65			9.31	104.31 ± 3.18		3.05
	Total		88.64 ± 2.70		3.04		94.15 ± 2.76			2.93		92.31 ± 6.28			6.81	102.69 ± 2.99		2.91

#### 3.2.5 Stability

The results of stability of GL-V9 and 5-O-glucuronide GL-V9 in Beagle dog plasma are shown in Table 3. The data indicated that GL-V9 was stable in dog plasma at room temperature for 23h, at 4°C after pretreatment for 119h, at the condition of -65~-90°C for 61 days and after 7 times freeze (-65~-90°C)-thaw (room temperature) cycles. 5-O-glucuronide GL-V9 was also demonstrated to be stable in dog plasma at room temperature for 22h, at 4°C after pretreatment for 120h, at -65 ~-90°C for 59 days and 6 times freeze (-65 ~-90°C)-thaw (room temperature) cycles.

**Table 3:** Stability results for GL-V9 and 5-O-glucuronide GL-V9 in Beagle dog plasma under different storage conditions (n = 6).

Storage Conditions	Conc. (ng/ml)	GL-V9				5-O-glucuronide GL-V9			
		Time /cycles	Mean ± SD (ng/ml)	Accuracy (%)	%CV	Time /cycles	Mean ± SD (ng/ml)	Accuracy (%)	%CV
Room temperature	1.5	23 h	1.39 ± 0.02	92.36	1.61	22 h	1.32 ± 0.03	87.82	2.35
	400		375.04 ± 4.89	93.76	1.30		356.2 ± 13.61	89.05	3.82
Post-treatment samples (4°C)	1.5	119 h	1.44 ± 0.01	95.71	0.60	120 h	1.51 ± 0.02	100.71	1.31
	400		388.4 ± 7.42	97.10	1.91		379.19 ± 14.33	94.80	3.78
Freeze -thaw Cycles	1.5	7	1.46 ± 0.02	97.31	1.47	6	1.55 ± 0.03	103.20	1.69
	400		394.75 ± 2.27	98.69	0.58		401.74 ± 3.73	100.44	0.93
Cryopreservation (-65°C~-90°C)	1.5	61 d	1.48 ± 0.07	98.67	4.52	59 d	1.57 ± 0.02	104.60	1.53
	400		387.07 ± 3.54	96.77	0.92		392.97 ± 11.86	98.24	3.02

In addition, GL-V9 and 5-O-glucuronide GL-V9 were shown to be stable in whole blood for 2.5 h, and the stock and working solutions of the analytes were also stable under the determined conditions (Table 4).

**Table 4:** Stability results for GL-V9 and 5-O-glucuronide GL-V9 in stock and working solution under different storage conditions (n = 6).

Solution	Conc.	GL-V9			5-O-glucuronide GL-V9		
		Conditions	Mean ± SD (%)	%CV	Conditions	Mean ± SD (%)	%CV
Working solution	LLOQ (5 ng/mL)	Room temperature (22 h)	99.22 ± 0.67	0.68	Room temperature (20 h)	100.56 ± 2.99	2.97
		-15°C~-30°C (71 d)	100.34 ± 1.39	1.38	-15°C~-30°C (71 d)	100.32 ± 2.18	2.17
	ULOQ (4000 ng/mL)	Room temperature (22 h)	99.98 ± 1.51	1.51	Room temperature (20 h)	98.73 ± 0.48	0.49
		-15°C~-30°C (71 d)	99.14 ± 0.79	0.80	-15°C~-30°C (71 d)	99.89 ± 1.30	1.31
Stock solution	1000 ng/mL	Room temperature (18 h)	101.25 ± 1.39	1.37	Room temperature (18 h)	99.10 ± 1.08	1.09
		-15°C~-30°C (85 d)	99.00 ± 1.43	1.44	-15°C~-30°C (85 d)	99.44 ± 0.90	0.90

Reproducibility, assessed by repeating measurements of the QC samples to assess precision and accuracy, met the acceptance criteria, indicating the samples could reinject in case of instrument interruptions or other reasons such as equipment failure.

### 3.3. Pharmacokinetic study

**Fig 4:**
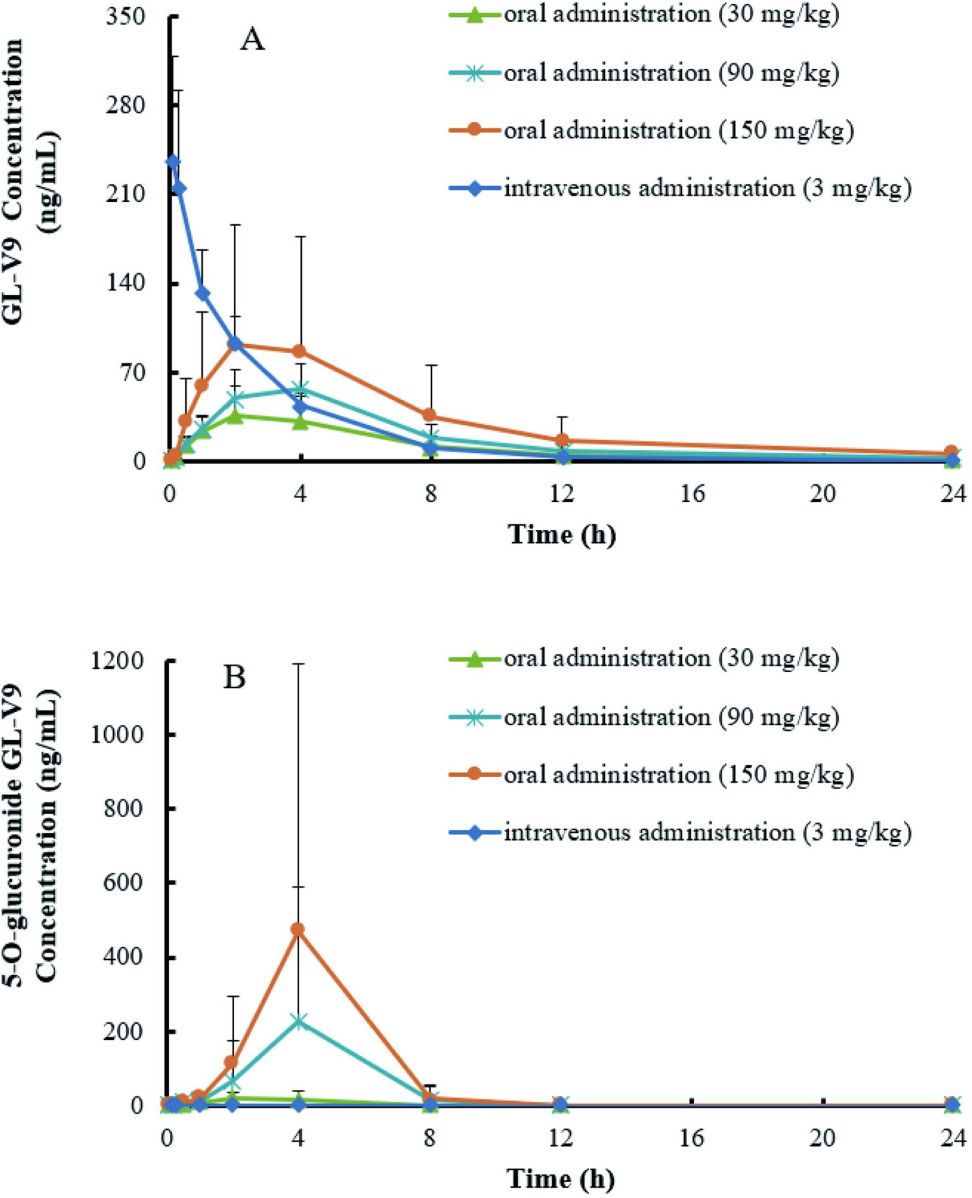
Mean concentration-time profiles of GL-V9 (A) and 5-O-glucuronide GL-V9 (B) in Beagle dog plasma after oral and intravenous administration. Each point indicates the mean ± SD from six animals.

For flavonoids, extensive phase II metabolism cannot be negligible because of the presence of hydroxy groups in the structure [10]. In this study, the plasma concentrations of GL-V9 and its glucuronide metabolites (5-O-glucuronide GL-V9) in Beagle dogs were detected by UPLC–MS/MS after administration. The mean plasma concentration-time profile is shown in Fig 4 and pharmacokinetic parameters calculated by a non-compartmental analysis are shown in Table 5.

**Table 5:** The pharmacokinetic parameters of GL-V9 and 5-O-glucuronide GL-V9 in Beagle dogs after administration of GL-V9 (*n* = 6, mean ± SD).

Analytes	Parameter	Units	Oral administration			Intravenous injection
			(30 mg/kg)	(90 mg/kg)	(150 mg/kg)	(3 mg/kg)
GL-V9	t_1/2_	h	3.65 ± 1.41	6.04 ± 1.77	7.06 ± 1.88	2.51 ± 0.755
	T_max_	h	2~4	2~4	2~4	0.083
	C_max_	ng/mL	39.2 ± 23.4	62.4 ± 19.6	92.5 ± 93.9	250 ± 88.0
	AUC_0~t_	(h·ng)/mL	250 ± 166	426 ± 158	763 ± 802	574 ± 142
	AUC_0~∞_	(h·ng)/mL	257 ± 168	446 ± 154	823 ± 838	579 ± 141
	Vd	L/kg	991 ± 826	2024 ± 1048	5022 ± 4639	18.9 ± 4.45
	MRT_0~∞_	h	5.43 ± 1.10	7.45 ± 1.22	9.02 ± 3.13	2.78 ± 0.500
	CL	L/(h·kg)	230 ± 237	220 ± 64.2	468 ± 405	5.43 ± 1.26
	F	%	4.35 ± 2.88	2.47 ± 0.915	2.65 ± 2.79	-
5-O-glucuronide GL-V9	T_max_	h	1~4	2~4	2~4	2
	C_max_	ng/mL	30.1 ± 22.4	228 ± 361	481 ± 711	1.68 ± 0.865
	AUC_0~t_	(h·ng)/mL	80.9 ± 74.2	870 ± 1401	1696 ± 2554	7.26 ± 5.83

t_1/2_: Elimination half-life; T_max_: time to peak concentration; C_max_: peak concentration; AUC: area under the concentration-time curve; V_d_: volume of distribution; MRT: mean residence time; CL: clearance; F: bioavailability; -: no data.

GL-V9 was rapidly absorbed and reached peak concentration (C_max_) at 2~4 h after oral administration and the C_max_ was 39.2 ± 23.4 ng/mL, 62.4 ± 19.6 ng/mL and 92.5 ± 93.9 ng/mL at the 30 mg/kg, 90 mg/kg and 150 mg/kg, respectively. The overall exposure (AUC_0-∞_) at low, medium and high dose were 257 ± 168 ng/ml*h, 446 ± 154 ng/ml*h and 823 ± 838 ng/ml*h, respectively. After intravenous administration, the plasma concentration decreased sharply and the half-life elimination time (t_1/2_) was 2.51 ± 0.755 h. The overall exposure (AUC_0-∞_) was 579 ± 141 ng/ml*h while the distribution volume (Vd) was 18.9 ± 4.45 L/kg, revealing that the residence time of GL-V9 in Beagle dogs was very short, suggesting that GL-V9 might be convert into its metabolites or quickly eliminated from the body. Previous literatures had reported the pharmacokinetic study after oral and pulmonary administration in rats [11]. It was shown that a twin peak was observed at 12h after administration in rat, which differed from the plasma concentration-time profile (Fig 4) in Beagle dogs after administration. The t_max_ was 0.31~0.77 h and 2~4 h after oral administration for rat and Beagle dogs, respectively, indicating GL-V9 displayed different absorption rate in rats and Beagle dogs. The clearance (CL) was 41.38 ± 8.84 L/h/kg in rats while the CL varied from 220 to 468 L/h/kg in Beagle dogs, which showed that the clear ability of GL-V9 varied in species.

Preparation and physiological factor may affect the bioavailability in species. Phylogenetically divergent between species may differ in handing of drugs in body involved in absorption, distribution, metabolism and elimination [16]. The solubility and permeability of the preparation may affect the degree of the adequate and reproducible absorption from the gastrointestinal tract (GIT) after oral administration [17]. The oral bioavailability of GL-V9 in dogs was 2.47%~4.35%. Previous study revealed that the oral bioavailability of GL-V9 in rats was 8.54%. The difference in bioavailability may be due to species, preparation solvent or other factors. Therefore, the difference in pharmacokinetic properties between species explains the need for this study.

The metabolite 5-O-glucuronide GL-V9 reached the peak at 1~4 h in Beagle dogs after oral administration, with the peak concentrations 30.1 ± 22.4 ng/mL, 228 ± 361 ng/mL and 481 ± 711 ng/mL at 30, 90 and 150 mg/kg, respectively, and the overall exposure (AUC_0-t_) was 80.9 ± 74.2 ng/ml*h, 870 ± 1401 ng/ml*h and 1696 ± 2554 ng/ml*h, respectively.

**Fig 5:**
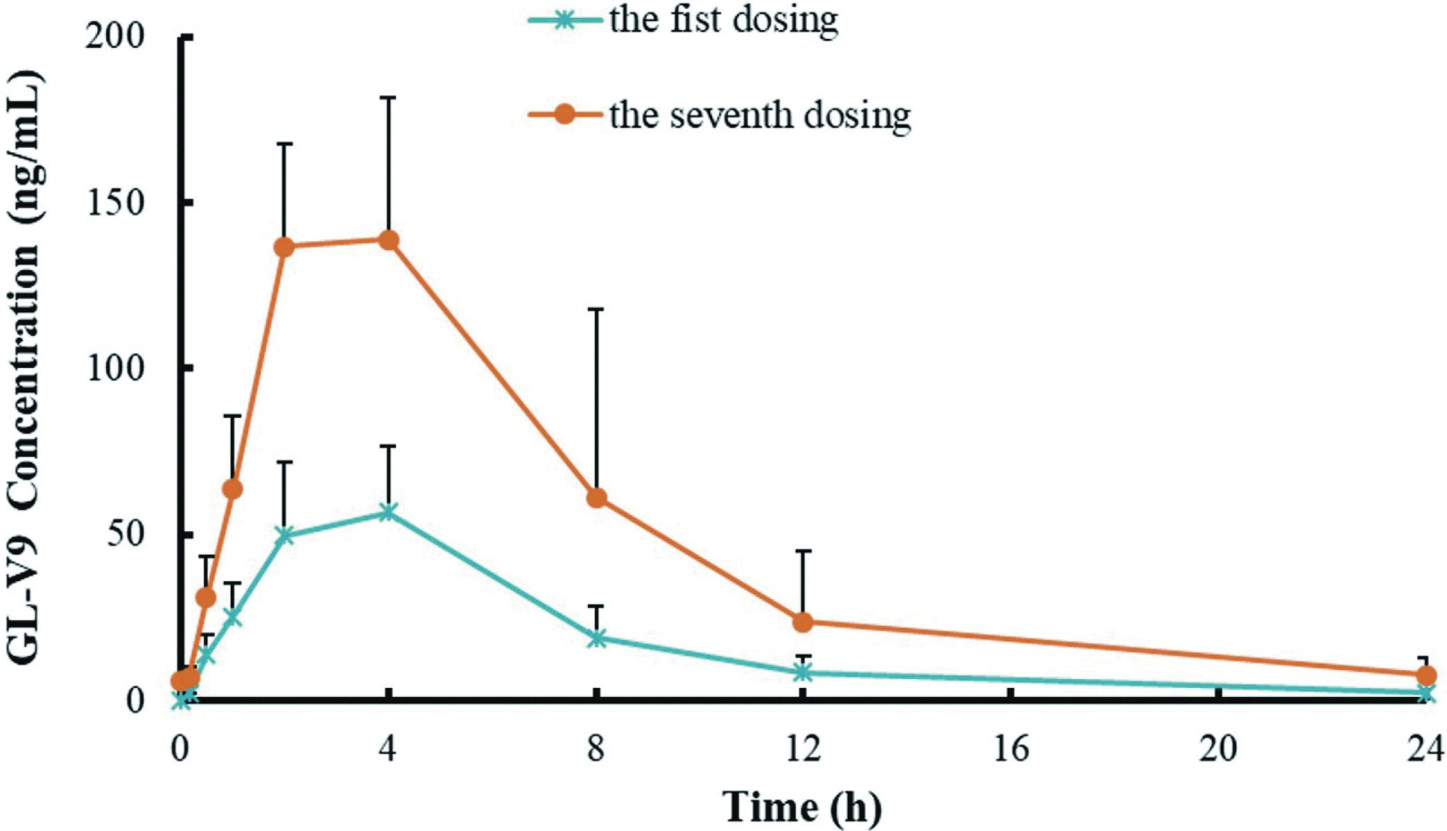
Mean concentration-time profiles of GL-V9 in Beagle dog plasma on the first dosing and the seventh dosing following once a day for one week by oral administration (90 mg/kg). Each point indicates the mean ± SD from six animals.

The pharmacokinetic profiles of GL-V9 after repeated oral administration (90 mg/kg) was also investigated, the plasma concentration vs. time profiles on the first dosing (first dose) and the seventh dosing (last dose) are shown in Fig 5. The plasma concentration of GL-V9 reached steady-state on the fifth day, and the trough concentration ranged from 5.16 ng/mL to 6.01 ng/mL. Additionally, the peak concentration (C_max_) of GL-V9 in Beagle dog plasma would be significantly increased on the last dose. Repeated administration might result in an increase in blood concentration but may not cause drug accumulation in Beagle dogs.

## 4. Conclusion

In this study, a highly selective, sensitive and rapid UPLC–MS/MS method for the determination of GL-V9 and its glucuronide metabolite (5-O-glucuronide GL-V9) was developed and validated. The method was effectively applied to evaluate the pharmacokinetic profile of GL-V9 and 5-O-glucuronide GL-V9 after oral and intravenous administration. GL-V9 was rapidly absorbed after administration and could be metabolited to 5-O-glucuronide GL-V9. The bioavailability of GL-V9 in Beagle dog was 2.47%~4.35% after oral administration. In addition, GL-V9 reached steady-state on the fifth day and the trough concentration was between 5.16 ng/mL and 6.01 ng/mL after repeated dosing at 90 mg/kg. In conclusion, the validated UPLC–MS/MS methods and the results obtained are expected to contribute to the further study and development of GL-V9.
